# Temporal and spatial characteristics of tumor evolution in a mouse model of oral squamous cell carcinoma

**DOI:** 10.1186/s12885-022-10256-5

**Published:** 2022-11-24

**Authors:** Yong Cao, Hao Dong, Guangyu Li, Huiping Wei, Cheng Xie, Yangjuan Tuo, Nian Chen, Dahai Yu

**Affiliations:** 1 Department of Oral and Maxillofacial Surgery, The People’s Hospital of Guangxi Zhuang Autonomous Region, 6 Taoyuan Road, Nanning, Guangxi 530021 P.R. China; 2 Research Center of Medical Sciences, The People’s Hospital of Guangxi Zhuang Autonomous Region, Guangxi Academy of Medical Sciences, Nanning, 530021 P.R. China; 3 College of Stomatology, Guangxi Medical University, 10 Shuangyong Road, Nanning, Guangxi 530021 P.R. China; 4 Department of Stomatology, The First Affiliated Hospital of Guangxi Medical University, 6 Shuangyong Road, Nanning, Guangxi 530021 P.R. China; 5 College& Hospital of Stomatology, Guangxi Medical University, Research Center for Craniofacial Deformity, Guangxi Key Laboratory of Oral and Maxillofacial Surgery Disease Treatment, No.10 Shuangyong road, Nanning, Guangxi 530021 P.R. China

**Keywords:** Oral squamous cell carcinoma (OSCC), Disseminated tumor cells (DTCs), Tumor evolution, Whole genome sequencing, Genetic heterogeneity, Tumor microenvironment

## Abstract

**Objectives:**

We aimed to elucidate the temporal and spatial characteristics of tumor evolution in an oral squamous cell carcinoma (OSCC) mouse model with higher burden of lymphatic metastasis through high-throughput sequencing.

**Methods:**

The OSCC model was established in 9 mice. DNA was extracted from the tumors of primary tongue lesions and disseminated tumor cells (DTCs) of submandibular gland lymph nodes and bone marrow, and then whole genome sequencing was performed. After bioinformatics analysis, somatic single-nucleotide variants (SSNVs) and copy number variations (CNVs) data were obtained. Based on SSNVs, clonal architecture and ancestor-descendant relationships among tumor cell subclones were elucidated.

**Results:**

A total of 238 tumor-related SSNVs with 120 high-frequency mutated genes were obtained from 36 samples of 9 mice by whole-genome sequencing. The number of unique SSNVs in the primary lesion, submandibular lymph node and bone marrow was greater than the number of shared SSNVs. Furthermore, the primary lesion-originated subclones, which were identified by SSNVs, were also detected in submandibular lymph nodes in the early stage of oral carcinogenesis. Moreover, at different histopathological stages, unique subclones were also identified in DTCs isolated from lymph nodes.

**Conclusion:**

Tumor heterogeneity is significant in primary tumor cells and disseminated tumor cells. OSCC cells probably disseminate to lymph nodes in the early stage of oral carcinogenesis. OSCC is characterized by polyclonal dissemination, and the evolutionary trajectory of DTCs is potentially dominated by the tumor microenvironment.

## Introduction

Oral squamous cell carcinoma (OSCC) is a common malignant tumor of the head and neck, which occurs in the oral cavity or oropharynx [1]. Invasion and metastasis of OSCC often result in treatment failure [2]. Up to 60% of patients with head and neck squamous cell carcinoma (HNSCC) can be expected to develop loco-regional recurrence [3] and 20–30% develop distant metastasis [4]. Recurrence is influenced by two main factors. On the one hand, the remaining tumor cells are insensitive to adjuvant therapy [5]. On the other hand, drug-resistant tumor cell subclones evolve after treatment [6], which is closely related to the continuous evolution of tumor cells under various selective pressures [7]. Tumor evolution typically follows a branched evolutionary model, resulting in the formation of different tumor subclonal groups and leading to tumor heterogeneity [7]. Tumor subclonal groups exhibit different abilities of growth, metastasis, invasion and resistance to the immune system, and present differences in sensitivity to radiotherapy and chemotherapy. Specific chemotherapeutic drugs often induce genetic mutations in tumor cell subgroups, such as those insensitive to the drugs. This feature leads to new drug-resistant tumor subgroup generation and proliferation, thus promoting tumor growth and recurrence. Therefore, tumor evolution represents a great challenge to treatment [8].

Tabatabaeifar et al. [9] performed an evolutionary analysis of primary, recurrent and metastatic tumor cells in OSCC patients using high-throughput sequencing. By drawing a phylogenetic tree of tumor cells, it was inferred that cells in recurrent tumors may be the same as disseminated cells in the early stage of carcinogenesis. Exome sequencing of HNSCC by Stransky [10] and Agrawal [11] revealed that 11 and 15%, respectively, of patients harbor Notch1 mutations. Notably, the Notch1 mutation rate reached 43% in OSCC patients [12].

Regarding tumor evolution, most studies on HNSCC have focused on primary tumors and cell lines [13], whereas researches on disseminated tumor cells from lymph node (LN) and bone marrow (BM) in the premalignant stage are limited. In our previous study, we established an OSCC mouse model with 4-nitroquinoline-1-oxide (4NQO) and found that cytokeratin (CK)-positive cells were detected in the submandibular LNs and BM during the histopathological stage of mucosal dysplasia by immunohistochemistry. Furthermore, homozygous deletion of exons in Tp53 and Rb1cc1 genes was determined in CK-positive cells, indicating that these cells were probably DTCs and early dissemination was a key characteristic in oral cancer [14]. However, the conclusion was based on the detection of CK-positive cells by immunohistochemistry and homozygous deletion in exons of the Tp53 and Rb1cc1 genes, and further evidence is needed. The enrichment of low-abundance tumor cells is one of the difficulties in the study of disseminated tumor cells. Immunomagnetic beads have obvious advantages in enriching lymph node and bone marrow disseminated cells [15]. CD45 is a pan-leukocyte marker, and depletion of CD45-positive cells is a critical step in enriching tumor cells [16]. EpCAM (epithelial cell adhesion molecule, CD326) has also been described as a reliable marke for the isolation of disseminated tumor cells in the bone marrow and lymph nodes of HNSCC [17]. Epithelial-derived disseminated tumor cells in bone marrow of breast cancer patients were harvested by CD326 immunomagnetic bead sorting [18]. At the same time, it is impossible to dynamically observe the evolution process of tumors clinically due to the difficulty in obtaining tumor cells and DTCs in the early stage of tumorigenesis. In this study, we performed whole genome sequencing of tumor cells derived from different tissues (tongue, submandibular LNs, and BM specimens) and distinct stages (mucosal dysplasia stage, OSCC stages with or without submandibular LN metastasis) using a 4NQO-induced OSCC mouse model. Tumor evolution was further explored based on the temporal and spatial characteristics of OSCC cells in the mouse model.

## Methods and materials

### Mouse model and sample collection

The animal experimental protocol was approved by the Animal Ethics Committee of Guangxi Medical University, China. To establish the OSCC mouse model, 70 BALB/c mice purchased from Guangxi Medical University Laboratory Animal Center were given drinking water containing 200 mg/l 4-NQO for 20 weeks [19]. From 22 to 36 weeks, 10 mice were sacrificed biweekly. Three hundred and twenty tissue samples, including tongue primary lesions (Pri), submandibular LNs, BM, and normal mucosa adjacent to the tongue primary lesions (Nor), were harvested from 70 animals.

### Histopathological staining and grouping

The collected tongue and lymph node tissue specimens were fixed in 4% paraformaldehyde and embedded in paraffin before being cut into 5 μm thick sections. The prepared sections were stained using H&E and Pan-CK following a routine staining procedure and examined by light microscopy. According to the World Health Organization (WHO) diagnostic criteria and histologic grading, histopathological stages were classified as normal mucosa, severe dysplasia, and squamous cell carcinoma. The histopathological stage of the mouse model was evaluated by two experienced pathologists. Pathologic analysis of tongue lesions was determined using hematoxylin & eosin (HE) staining. Lymph node metastasis was confirmed by the detection of clumped tumor cells according to pan-CK staining (Boster, Wuhan, China, NBP2–48300). Three mice for each group, including severe dysplasia (designated the “D” group: mouse number as 1–3), oral cancer without lymph node metastasis (designated the “T” group: mouse number as 4–6), and oral cancer with lymph node metastasis (designated the “M” group: mouse number as 7–9), were selected for subsequent experiments.

### DTCs enrichment by CD45/CD326 magnetic-activated cell sorting (MACS)

Bone marrow mononuclear cells were isolated from bone marrow aspirate using the density gradient centrifugation method [14]. Single-cell suspensions of lymph nodes were prepared following reference [20]. The derived cells were incubated with 100 μL CD45 (130–052-301, Miltenyi Biotec, Germany) immunomagnetic beads at 4 °C for 30 min. 10 mL buffer was added to suspend the above cells and centrifuged at 300 g, 4°Cfor 10 min. The sediments were resuspended with 5 mL buffer and applied onto the prepared LS Column attached with Midi MACS separator. Collect unlabelled CD45 negative cells which pass through the column with the buffer for further enrichment. Above collected CD45 negative cells were centrifuged at 300 g, 4 °C for 10 min and resuspended with 400 μL buffer. 100 μL CD326 (130–105-958, Mitanni Biotec, Germany) immunomagnetic beads were added to incubated for 30 min at 4 °C. Remove the column from the separator and immediately flush out the magnetically labelled CD326 positive cells by firmly applying the plunger with 5 mL buffer. The cells were then washed 3 times with buffer to remove remanent immunomagnetic beads.

### Flow cytometry

Cells were concentrated by centrifugation for 4 min at 1200 g. After supernatant removal, the cells were resuspended and stained with 10 μL CD326 antibody conjugated to phycoerythrin (PE) (130–102-234, Miltenyi Biotec, German) yand incubated in the dark at room temperature for 12 min. Cell pellets were resuspended in 500 μL PBS and counted by flow cytometry using a BD FACSCantoTM II apparatus (Becton Dickinson, San Jose, CA, USA).

### DNA extraction, library construction, and sequencing

DNA was extracted from fresh frozen tissue of mouse tongue primary lesions (Pri) and normal mucosa adjacent to tongue primary lesions (Nor) using a Qiagen QIAamp DNA Kit (51,306, Germany Qiagen). Genomic DNA and whole genome amplification of DTCs from LNs and BM collected from fresh animal tissue were performed using the Qiagen Single Cell REPLI-g Kit (150,343, QIAGEN, Germany).

### Bioinformatics processing

Normal mucosa adjacent to tongue primary lesions (Nor) served as the germline control. Clean reads were mapped to the mm10 mouse genome reference using the Burrows-Wheeler Aligner (BWA) [21]. Duplicate reads were marked and removed by Picard-Tools-v1.115 (http://broadinstitute.github.io/picard). Somatic single nucleotide variants (SSNVs) were called by Sentieon TNseq. The obtained SSNVs were filtered using GATK Variant Filtration, and ANNOVAR (ANNOtate VARiation) was used for variant annotation [22]. Copy number variations (CNVs) were detected using the mutation analysis software Control-FREEC [23] and then analyzed by GISTIC 2.0 ref. (Genomic Identification of Significant Targets in Cancer) [24] using a q-value cutoff of 0.05 to assess significant mutations of gains and losses. High-frequency tumor SSNVs were retained after removing the low-frequency SSNVs with a cancer cell fraction (CCF) < 5%. By matching to the Catalogue of Somatic Mutations In Cancer (COSMIC)_v90 data, high-frequency SSNVs related to human cancers were obtained.

### Phylogenetic tree construction and clonal analyses

Tumor cell ploidy and tumor purity were evaluated using ABSOLUTE software [25], and CCF somatic mutations were determined by PyClone [26]. Tumor subclones were acquired by clustering of the CCF in tumor cells with different tissue resources. Clone trees were constructed by ClonEvol [27] by searching for the top-ranking lineage trees, and the clonal evolution of tumor cells for each sample was established. Considering the sequencing depth and tumor purity in samples, the phylogenetic tree was constructed according to the following principles: (1) Tumor subclones shared by the primary lesion and metastatic tissues were defined as the trunk of the phylogenetic tree; (2) The “Most Recent Common Ancestor (MRCA)” of the primary and metastatic tumors harbored tumor-derived mutations referring to the COSMIC database; (3) The evolutionary relationship of the shared subclones was determined by primary tumor cells; (4) The unique subclones of primary or metastatic tumor cells were the branches in tumor evolution, and the evolutionary trajectory was constructed according to the genetic distance among subclones.

## Results

### OSCC formation and local lymph node enlargement

**Fig. 1:**
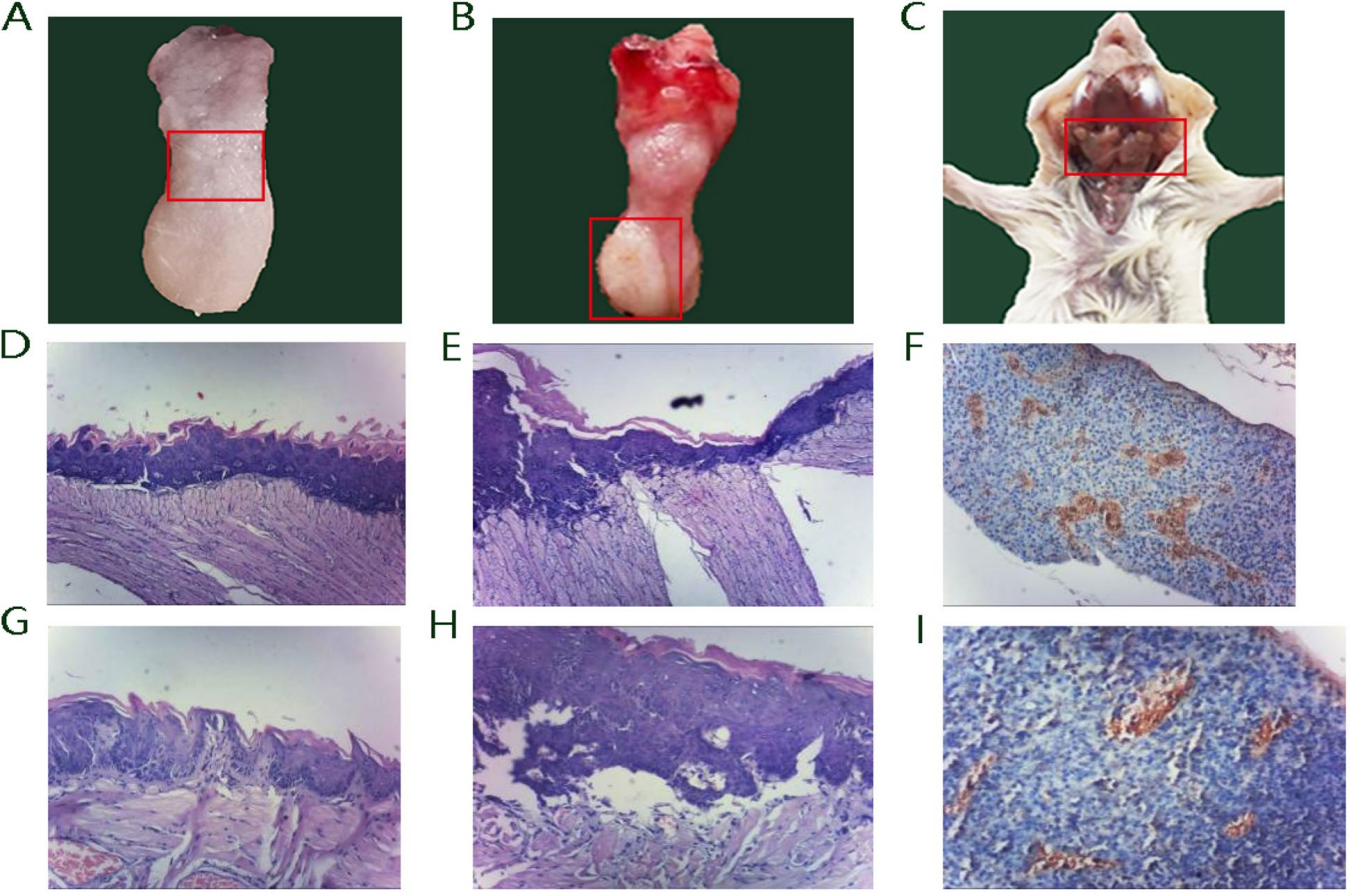
Pathological evidence of tumor formation and local lymph enlargement induced by 4NQO. **A** Dysplasia; **B** Tongue cancer; **C** Lymph node metastasis; **D** Abnormal hyperplasia (100×); **E** Tongue cancer (100×); **F** Lymph node metastasis (100×); **G** Abnormal hyperplasia (200×); **H** Tongue cancer (200×); **I** Lymph node metastasis (200×)

After 20 weeks’ 4NQO treatment and 2 weeks’ observation, tumor formation and local lymph enlargement were observed. In the early stage, moderate to severe dysplasia of the tongue mucosa occurred, where the back of the tongue could be seen to be rough and slightly tough to the touch (Fig. 1A). At 28 weeks, a whitish thickened patch covered almost the entire surface of the right tongue (Fig. 1B). Moreover, enlarged lymph nodes were observed (Fig. 1C). The histopathological stages of the mouse model were determined by hematoxylin & eosin (HE) staining and pan-CK staining. HE staining of tongue mucosa with severe dysplasia showed that the prickle cell layer of tongue dorsal mucosa was thickened, and a few karyokinesis, but the basement membrane was intact (Fig. 1D, G). HE staining of highly differentiated squamous cell carcinoma showed complete disorder of cell stratification, frequent karyokinesis, and occasionally cornified pearl formed in the center of the cancer nest (Fig. 1E, H). A large number of cytokeratin positive tumor cells in flaky distribution can be seen in the tumescent lymph nodes (Fig. 1F, I). After histopathological examination,17 of 70 mice had severe dysplasia, 13 of them suffered from oral squamous cell carcinoma without lymph node metastasis, and 4 of them suffered from oral squamous cell carcinoma with lymph node metastasis.

### DTCs in a mouse OSCC model can be enriched by CD45/CD326

**Fig. 2:**
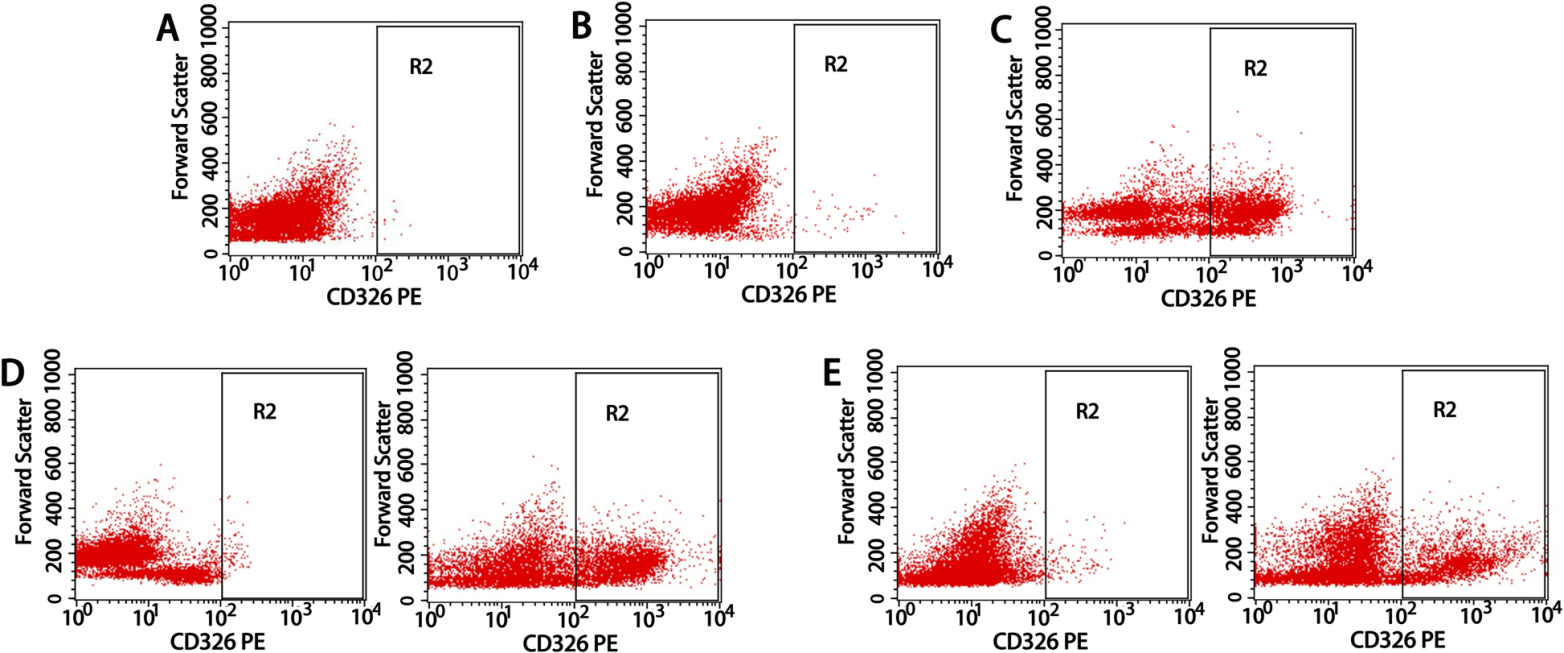
Flow cytometry results of Single cell suspension before and after MACS. **A** Single-cell suspension from a normal lymph node before MACS. **B** Single-cell suspension from normal bone marrow before MACS. **C** Single-cell suspension from tongue cancer before MACS. **D** Single cell suspension of lymph node before and after sorting with CD45/CD326 immunomagnetic beads (mouse 1). **E** Single-cell suspension of bone marrow monocytes before and after sorting with CD45/CD326 immunomagnetic beads (mouse 1)

Using flow cytometry analysis, CD326 was expressed at very low levels in normal lymph node and bone marrow cells (Fig. 2A-B) but highly expressed in tongue cancer tissue (Fig. 2C). After MACS sorting, the proportion of DTCs in lymph node and bone marrow Single-cell suspension of mouse 1 was increased into 30.22 and 29.47% respectively. (Table 1, Fig. 2D-E). This suggests that CD326 magnetic bead sorting can be used to enrich DTCs in lymph nodes and bone marrow. The average purity of primary tumor of oral squamous cell carcinoma in mice was 33.06% ± 5.66% (Table 2), which was similar to the results of Zandberg [25], suggesting that the cell composition in the OSCC mouse model shared high similarity to human OSCC. It should be noted that the estimated average purity of disseminated tumor cells in bone marrow and lymph node was 32.86 ± 3.72 and 38.09% ± 7.66% respectively (Table 2), which is approximate to the results of flow cytometry (26.51% ± 3.58%; 32.25% ± 3.73%, Table 1). This consistency verified the feasibility and reliability of MACS for enriching DTCs.

**Table 1:** The positive rate of CD326 cells in lymph nodes and bone marrow single cell suspensions

Mouse number	Lymph node		Bone marrow	
	Before sorting	After sorting	Before sorting	After sorting
1	0.14%	30.22%	2.31%	29.47%
2	0.9%	36.27%	1.15%	23.92%
3	0.88%	29.31%	1.24%	31.61%
4	0.67%	31.28%	0.73%	26.72%
5	1.3%	30.66%	0.87%	20.61%
6	0.74%	31.22%	1.4%	27.51%
7	1.28%	40.21%	1.55%	30.21%
8	0.75%	32.66%	0.93%	23.41%
9	1.03%	28.43%	0.77%	25.14%
$\overline{x}$±S	0.85% ± 0.35%	32.25 ± 3.73%	1.22% ± 0.49%	26.51 ± 3.58%

**Table 2:** Tumor ploidy and tumor purity

Sample	Purity	Ploidy
BMDTCs1	29.40%	1.91439197
BMDTCs2	29.38%	1.79430189
BMDTCs3	34.60%	1.91785198
BMDTCs4	29.51%	1.74682886
BMDTCs5	37.00%	1.92126650
BMDTCs6	29.45%	1.94589481
BMDTCs7	31.51%	2.00604670
BMDTCs8	37.48%	1.98312967
BMDTCs9	37.40%	1.93134884
$\overline{x}$±S	32.86 ± 3.72%	1.91 ± 0.84
LNDTCs1	29.89%	1.37358258
LNDTCs2	30.24%	1.75200212
LNDTCs3	28.23%	2.06352792
LNDTCs4	44.13%	0.99355114
LNDTCs5	50.61%	2.04485691
LNDTCs6	37.73%	2.03978878
LNDTCs7	36.53%	1.94539522
LNDTCs8	44.24%	2.03353282
LNDTCs9	41.18%	2.02499590
$\overline{x}$±S	38.09% ± 7.66%	1.81 ± 0.38
Pri1	31.49%	2.13562497
Pri2	29.57%	2.15996195
Pri3	35.76%	2.11567815
Pri4	26.69%	1.94782736
Pri5	27.53%	2.20524772
Pri6	32.52%	2.12615989
Pri7	35.98%	2.08285671
Pri8	32.56%	2.14081562
Pri9	45.48%	2.13503622
$\overline{x}$±S	33.06% ± 5.66%	2.12 ± 0.71

### Genetic heterogeneity between primary lesions and DTCs

**Fig. 3:**
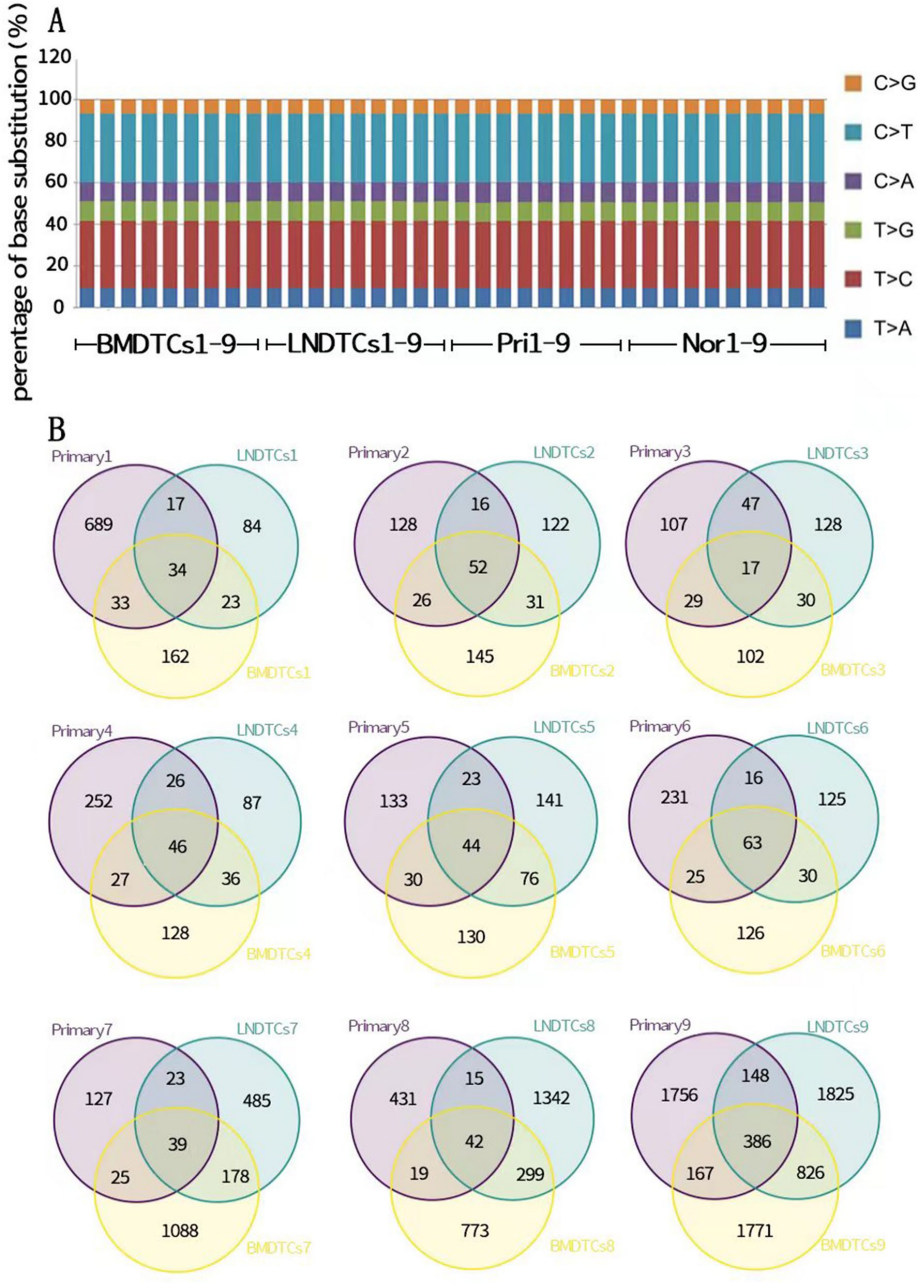
List of SSNV mutation patterns and SSNVs from different tissues were characterized by Venn diagrams in 9 mice. **A** Summary of 36 samples SSNV mutation patterns LNs: lymph nodes, BM. **B** Tumor SSNVs from different tissues were characterized by Venn diagrams in 9 mice. The purple circle represents SSNVs identified in the primary lesion, the blue circle represents SSNVs identified in DTCs isolated from lymph nodes, and the brown circle represents SSNVs in DTCs isolated from bone marrow. Numbers in the circle indicated the number of SSNVs. LNs: lymph nodes, BM: bone marrow, DTCs: disseminated tumor cells, Nor: Adjacent tissue, Pri: Primary tumor

Whole-genome sequencing was performed on 36 samples, of which 9 were adjacent normal mucosa, tongue primary lesions, LN DTCs, and BM DTCs. Numerous C > T and T > C conversion mutations were noted in the OSCC mouse models (Fig. 3A). The number of SSNVs from different tissue sources in each mouse was plotted using Venn software, and the numbers of unique SSNVs among primary lesions, LNs and BM were all greater than those of shared SSNVs, suggesting that tumor cells from different tissues in the same mouse exhibited significant genetic heterogeneity in SSNVs (Fig. 3B). Using the COSMIC database, human cancer-related SSNVs were isolated from the SSNVs residing in the exon regions in all samples, and 238 tumor-related SSNVs were obtained, of which 120 were high-frequency mutated genes with a mutation frequency ≥ 5%. The top 10 most frequently mutated genes were as follows: Sirpb1a, Cdh11, Smarca4, Fat1, Gata3, Notch1, Usp32, Cdk12, Cic, and Creb312. Fat1- and Notch1-harboring SSNVs were associated with OSCC in humans, revealing similarities between human OSCC and the mouse OSCC model.

### Chromosomal amplifications and deletions in mouse OSCC

**Fig. 4:**
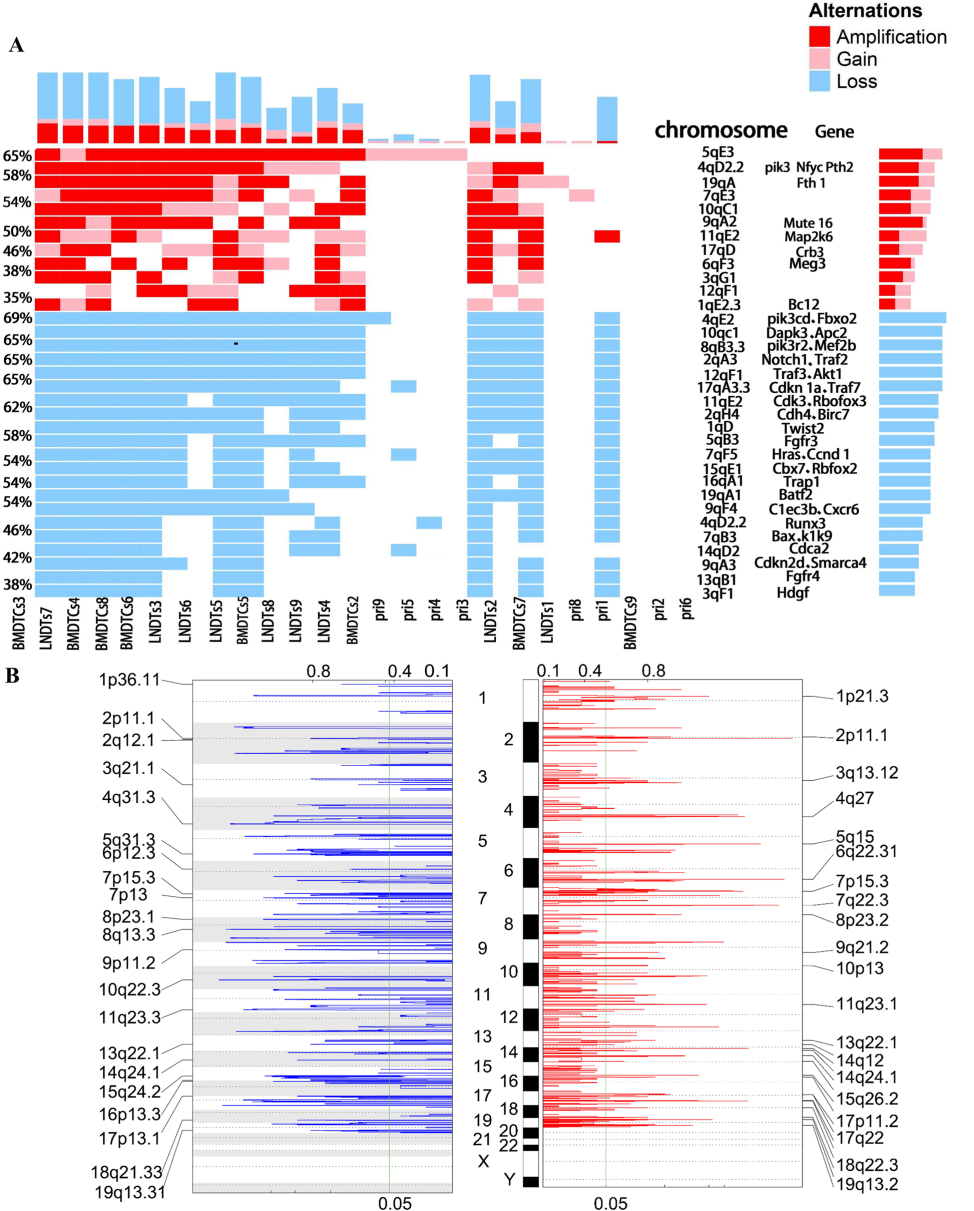
Heatmap of CNVs with the significantly amplified/deleted cytoband and gains and losses of copy number. **A** The red square indicates the cytoband with amplification/gain, and the blue square indicates the cytoband with deletion. The y-axis shows the mutation percentage in the corresponding chromosomal region. The x-axis represented sample name. The “Genes” column lists mutated genes related to human cancers located in chromosome fragments. **B** Gains and losses of copy number identified by GISTIC 2.0.A: Copy number gains identified in all tumor cells (primary and DTCs). B. Copy number deletions identified in all tumor cells (primary and DTCs)

Twelve chromosome amplification bands and 21 deletion bands were identified by GISTIC 2.0 analysis (Fig. 4B). The significant copy number amplifications were mainly located in regions of 3qG1, 4qD2.2, 5qE3, 7qE3, 9qA2, 10qC1, and 19qA, whereas deletions were mainly located in regions of 1qD, 2qA3, 3qF1, 4qE2, 7qB3, 7qF5, 8qB3.3, 9qA3, 11qE2, 12 pF1, 14qF, and 17qA3.3. These regions contained tumor-related genes, such as Muc16, Pik3, and Bcl2 in amplification regions and Hras, Notch1, Apc2, Ccnd1, Batf2, Dapk3, Smarca4, Traf2, Traf3, Cdk3, and Cdh4 in deletion regions (Fig. 4A). Deletions in Hras, Notch1, and Ccnd1 were common mutations in human OSCC, and these mutations were also found in the OSCC mouse model, further confirming the similarity in CNVs between human OSCC and this model. The CNVs were mainly concentrated in DTCs rather than in primary tumor cells. This finding might be due to the uneven amplification of the DTC whole genome, which increased the chance that CNVs were detected in DTCs after high-throughput sequencing.

### Characteristics of tumor clonal evolution from normal mucosa status to lymphatic metastasis stage

**Fig. 5:**
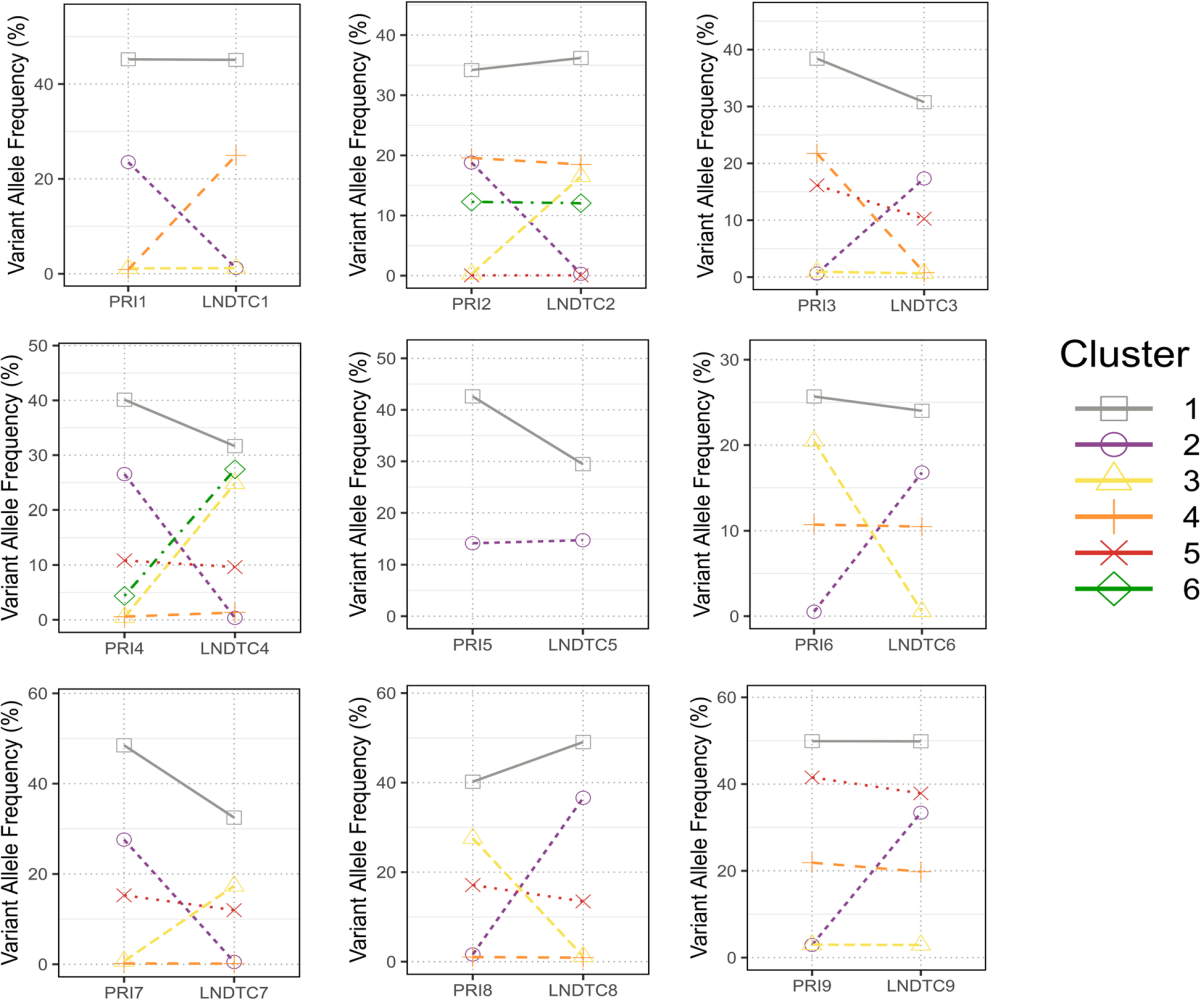
Average value of clustering between samples between 9 mice. Paired comparison of the mean distribution of Variant Allele Frequency subsets between primary tongue tumor cells and lymph node metastatic tumor cells in 9 mice: 1–3 dysplastic mice, 4–6 mice without lymph node metastasis, 7–9 mice with lymph node metastasis. Variant Allele Frequency subgroups were obtained by clustering the CCF values of tumor cells in different parts of each mouse. The Variant Allele Frequency subgroups of each mouse correspond to a specific color and shape

**Fig. 6:**
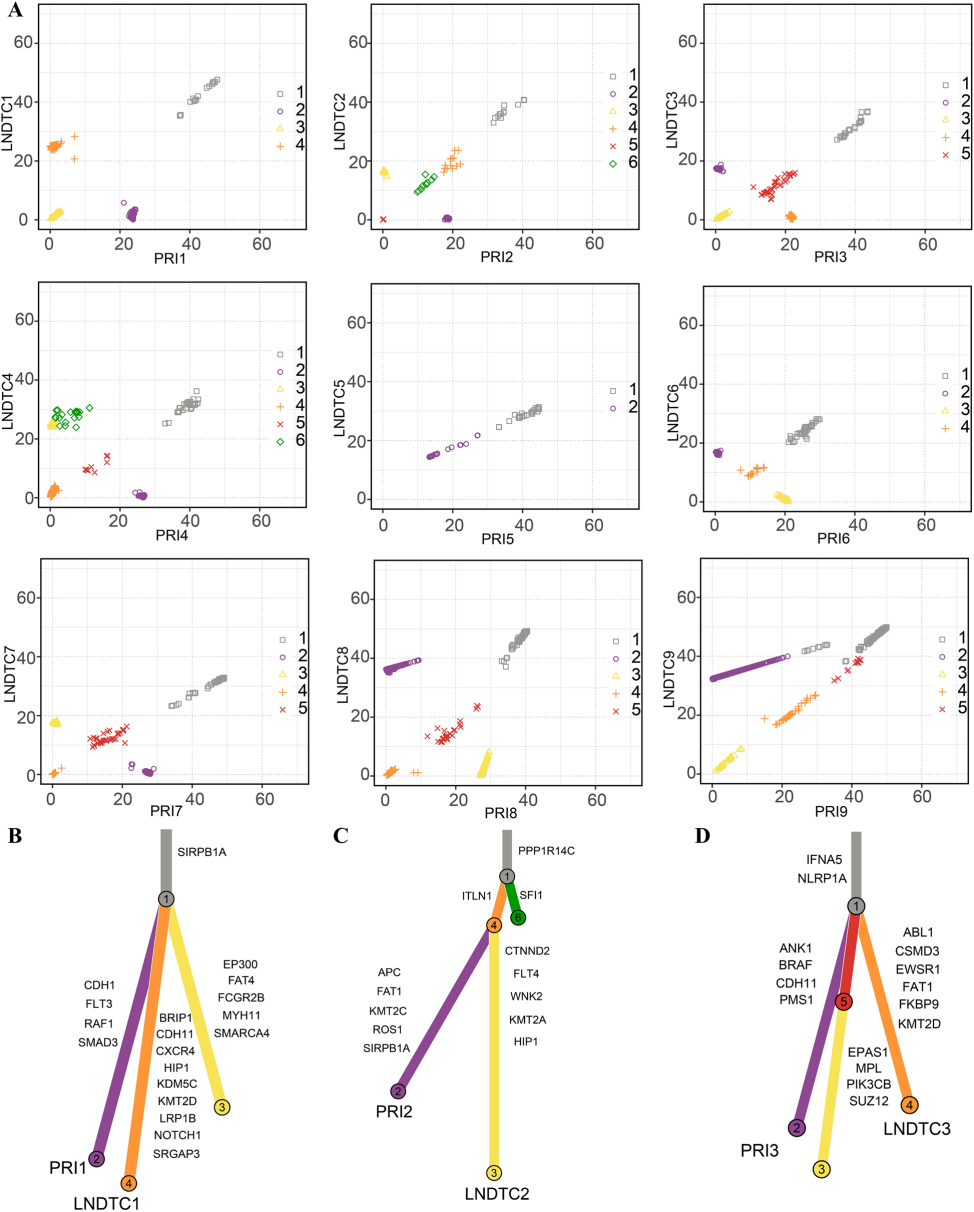
Subclonal architecture of 9 mice and relationships between subclones were revealed by phylogenetic trees in three mice. **A** Two-dimensional scatter plots show the CCF of the mutations among different tumor cells. Each subclone was acquired by clusting of the CCF in tumor cells from different organs. **B**-**D** Phylogenetic trees of mouse1–3: Branch length was proportional to the number of substitution mutations in each subclone. The trunk branch represents subclones originating from the primary lesion, and the subbranch represents unique subclones from different tissues. Tissue resources were annotated at the end of the branch, and oncogenic genes with driver mutations were annotated on the side of the branch

PRI: tongue primary tumor cells; LNDTC: lymph node disseminated tumor cell samples and lymph node metastases tumor cells.

To define the clonal architecture in each mouse, it is necessary to compare the individual mutated CCFs between primary lesions and DTCs using two-dimensional plots. Tumor subclones were identified by clustering mutations exhibiting shared CCF. Shared subclones between primary tumors and DTCs (mouse1–6) were identified in mice, implying that tumor dissemination occurred in the early stage of carcinogenesis (Fig. 5). Taking mouse 1 as an example, subclone 1 was found in both primary tumor and DTCs from LNs (Fig. 5). Interestingly, unique subclones between primary tumors and DTCs were identified in mouse1–9, which resulted in tumor heterogeneity between primary and metastatic sites of OSCC. (Fig. 6A). Phylogenetic analysis was employed to evaluate the ancestor-descendant relationship among clones. The phylogenetic tree showed that tumor subclones in LN in the same mouse (mouse1, 2, 3, Fig. 6B-D) simultaneously originated from the primary tumor and eventually formed distinct branches, indicating that the evolutionary trajectory of DTCs was affected by the tumor microenvironment. During the evolution of tumor cells, driver mutation occurred in a large number of oncogenic genes, including Smarca4, Cxcr4, Ctnnd2 and Ank1 (Fig. 6B-D).

## Discussion

Recently, several studies have revealed genetic intratumor heterogeneity (ITH) and complex evolution patterns in various tumors using next-generation sequencing [28]. Genetic ITH of clonal evolution can lead to treatment failure and drug resistance [29]. Hence, understanding clonal evolution from a normal cell status to the primary carcinoma stage and subsequent metastatic stage is the foundation for cancer prevention and treatment. To our knowledge, this study is the first report characterizing OSCC clonal evolution from the normal mucosa status to the primary carcinoma stage and metastatic stage in a mouse model.

CD326 immunomagnetic bead sorting was frequently used for enriching tumor cells in peripheral blood of lung cancer and esophageal cancer [30, 31]. The detection rate of tumor cells in peripheral blood was improved from nearly undetectable to more than 24.14% by using CD45 and CD326 immunomagnetic beads [32]. Our study showed that the proportion of DTCs increased from 0.85 to 32.5% in lymph nodes and from 1.22 to 26.51% in bone marrow. CD45/CD326 immunomagnetic beads technique in enriching low-abundance OSCC tumor cells in our study performed well when compared with other studies, providing a new feasible method for the enrichment of oral cancer cells.

We found that mouse OSCC has *C > T* and *T > C* mutation rates similar to those of human OSCC [33]. Mutations and deletions of the Fat1, Notch1, Casp8, Hras, and Ccnd1 genes in human OSCC were also found in an OSCC mouse model according to the analysis of SSNVs and CNVs. Genetically, these results indicate that human OSCC can be mimicked by the OSCC mouse model, which is consistent with our previous histopathology study [34].

The SSNV Venn diagram showed that tumor cells from different tissues exhibit significant heterogeneity in the same mouse. This result is similar to that reported by Zandberg et al. study [25], namely, tumor cells in different regions of OSCC primary lesions exhibit heterogeneity.

With the popularization of next-generation sequencing technology, evolutionary analysis of primary tumor cells and DTCs has become a new method from which the origin of DTCs can be inferred [35]. Due to the possible bias of cell copy number induced by whole genome amplification, SSNV data were used for cell subclonal analysis in our study.

Recent studies suggest that in the severe dysplasia and nonmetastatic stages of OSCC (clinically equivalent to precancerous and CN0 stages), tumor subclones that originated from primary lesions were identified in DTCs in LNs and BM by whole genome sequencing. As previously mentioned, deletions or mutations in the Hras, Notch1, Apc and Fat1 genes have been identified in human OSCC. A similar phenomenon of these genes is also found in DTCs in an OSCC mouse model with severe dysplasia and nonmetastatic stages. These cells might be derived from primary lesions and disseminate in the early stage. In Tabatabaeifar’s study [9], samples were derived from patients with OSCC, which made it impossible for them to determine the specific pathological stage of DTC dissemination. In the current study, specimens were obtained from a mouse model under different stages of oncogenesis, which allowed us to study the tumor evolution of OSCC before LN metastasis. Using these specimens, tumor subclones originating from primary tumors were identified in DTCs, which provided direct evidence for the early spread of OSCC. As a result of early spread, DTCs in LNs and BM undergo parallel evolution along with primary tumor cells. Hence, significant heterogeneity in both primary tumors and DTCs was observed. SMARCA4 mutation directly results in the inability of the SWI/SNF complex, promoting early metastasis of lung cancer [36]. High expression of CXCR4 in breast cancer is associated with early distant metastasis and bone metastasis [37]. The use of CXCR4 inhibitor can effectively inhibit the early metastasis of triple negative breast cancer [38]. In addition, the early spread of breast cancer is related to the inactivation of RUNX3 gene caused by hypermethylation of promoter and protein mislocation [39]. There are rare reports on the mechanism of early dissemination of head and neck cancer. During the current study, we also found mutations of Smarca4, Runx3 and Cxcr4 in early disseminated tumor cells. We speculated that the early dissemination and metastasis of OSCC may be related to the driving mutations such as SMARCA4, CXCR4 and RUNX3, which provides a theoretical basis for the future study on the mechanism of early spread and metastasis of oral cancer.

It has been reported that polyclonal dissemination to LNs exists in esophageal squamous cell carcinoma [40], breast cancer [41] and lung cancer [42]. When tracking the evolutionary trajectory of tumor clones in the mouse model, we found that multiple subclones originating from primary lesions were present in DTCs in LNs and BM, indicating that polyclonal dissemination to LNs might also occur in OSCC. Furthermore, the spreading ability of OSCC cells may be acquired in the early stage of tumor evolution. Due to chromosomal variation, malignant cells, which are transformed from normal epithelial cells, might acquire metastatic ability in the early stage of tumor evolution. This notion contradicts previous theory that only advanced tumor cells possess metastatic ability.

Our results reveal different subclones with various tumor-driving mutations in DTCs, which are isolated from LNs in the same mouse, indicating that selective pressure may change the evolutionary trajectory of tumor cells in microenvironments of LNs. This finding is consistent with Böhrnsen et al.’s study [43]. These researchers cocultured human bone marrow stem cells (BMSCs) with HNSCC PCI-13 cells and found that BMSCs could inhibit the epithelial mesenchymal transformation (EMT) of HNSCC cells. Another study showed that coculture of an OSCC cell line with BMSCs enhanced tumor invasion but inhibited tumor proliferation [44]. In our previous study, we also showed that LN microenvironment exerts different proliferative and inhibitory effects on primary tumor cells [45]. Obviously, there are several limitations in our study. Firstly, the tumor size from the primary lesion in the OSCC mouse model was too small to obtain samples from multiple tumor regions, hinders us from studying the ITH in different tumor regions. Secondly, limited by the sequencing depth, the mutation loci we found needs to be further verified. Thirdly, the mechanism of high-frequency gene mutation and early metastasis found in this study also needs to be further elucidated. In the future, researchers could consider obtaining samples from multiple regions in the primary and metastatic lesions and then performing ultradeep sequencing to analyze the mechanism of tumor heterogeneity.

In summary, our study is the first report the evolutionary relationship of primary tumor cells and DTCs isolated from LNs, based on investigations in an OSCC mouse model at different pathological stages by high-throughput sequencing. The highlights of this study include the following points: significant tumor heterogeneity is noted in primary lesions and DTCs in the OSCC mouse model. Based on clonal evolutionary analysis, OSCC cells are characterized by early-stage dissemination and polyclonal dissemination. Different tumor microenvironments may change the evolutionary trajectory of DTCs.

## Data Availability

The datasets generated and analyzed during the current study are available in the Sequence Read Archive (SRA) database (PRJNA837417, https://www.ncbi.nlm.nih.gov/sra/PRJNA837417).
